# Neandertal Humeri May Reflect Adaptation to Scraping Tasks, but Not Spear Thrusting

**DOI:** 10.1371/journal.pone.0040349

**Published:** 2012-07-18

**Authors:** Colin N. Shaw, Cory L. Hofmann, Michael D. Petraglia, Jay T. Stock, Jinger S. Gottschall

**Affiliations:** 1 PAVE Research Group, Department of Archaeology & Anthropology, University of Cambridge, Cambridge, United Kingdom; 2 Neuromotion Laboratory, Department of Kinesiology, The Pennsylvania State University, University Park, Pennsylvania, United States of America; 3 Research Laboratory for Archaeology and the History of Art, School of Archaeology, University of Oxford, Oxford, United Kingdom; 4 McDonald Institute for Archaeological Research, Department of Archaeology and Anthropology, University of Cambridge, Cambridge, United Kingdom; University of Kansas, United States of America

## Abstract

Unique compared with recent and prehistoric *Homo sapiens*, Neandertal humeri are characterised by a pronounced right-dominant bilateral strength asymmetry and an anteroposteriorly strengthened diaphyseal shape. Remodeling in response to asymmetric forces imposed during regular underhanded spear thrusting is the most influential explanatory hypothesis. The core tenet of the “Spear Thrusting Hypothesis”, that underhand thrusting requires greater muscle activity on the right side of the body compared to the left, remains untested. It is unclear whether alternative subsistence behaviours, such as hide processing, might better explain this morphology. To test this, electromyography was used to measure muscle activity at the primary movers of the humerus (*pectoralis major* (PM), *anterior* (AD) and *posterior deltoid* (PD)) during three distinct spear-thrusting tasks and four separate scraping tasks. Contrary to predictions, maximum muscle activity (MAX) and total muscle activity (TOT) were significantly higher (all values, p<.05) at the left (non-dominant) AD, PD and PM compared to the right side of the body during spear thrusting tasks. Thus, the muscle activity required during underhanded spearing tasks does not lend itself to explaining the pronounced right dominant strength asymmetry found in Neandertal humeri. In contrast, during the performance of all three unimanual scraping tasks, right side MAX and TOT were significantly greater at the *AD* (all values, p<.01) and *PM* (all values, p<.02) compared to the left. The consistency of the results provides evidence that scraping activities, such as hide preparation, may be a key behaviour in determining the unusual pattern of Neandertal arm morphology. Overall, these results yield important insight into the Neandertal behavioural repertoire that aided survival throughout Pleistocene Eurasia.

## Introduction

For Neandertals living in the variable, and at times cold, climate of the Late Pleistocene high levels of energy expenditure and meat consumption were essential to survival [1]–[4]. While isotopic evidence suggests a life requiring the hunting or scavenging of large game [3], it remains unclear which habitual activities the fossilized skeletal remains of Neandertals and associated archaeological materials reflect. Numerous skeletal traits differentiate the postcranial skeleton of European and Levantine Neandertals from modern [5]–[7] and also prehistoric *Homo sapiens* [8]–[16]. Two particular characteristics have received considerable attention; pronounced humeral diaphysis strength asymmetry and anteroposteriorly strengthened humeral diaphyseal shape [5], [17]–[20]. In particular, humeral bilateral asymmetry for cross-sectional area, and torsional and average bending rigidity, appear exceptionally high in Neandertals (averaging 24–57%) compared to skeletal samples of modern Holocene *H. sapiens* (averaging 5–14%) [5]. While Upper Paleolithic *H. sapiens* also display relatively high levels of humeral bilateral asymmetry [14], the only living human groups with similar morphological characteristics are tennis players and cricketers (averaging 28–57% asymmetry for the same measurements) who habitually and asymmetrically load their upper limbs [5], [21].

An influential hypothesis by Churchill and colleagues [17], which has been cited broadly [22]–[32], argues that regular close-range hunting with thrusting spears produced the Neandertals pronounced right-dominant humeral strength asymmetry and highly anteroposteriorly strengthened diaphyses. The authors suggested that during underhand spear thrusting the dominant limb (positioned towards the back) generates the majority of the force, while the non-dominant limb (positioned towards the front) serves to guide and stabilize the spear [17]. Considerable experimental evidence [33]–[35] supports the idea that long bone diaphyses respond to increased forces by structurally augmenting their mass in the principal planes of deformation [36], [37]. Thus, asymmetric loading associated with spear thrusting was proposed to cause bilaterally asymmetric adaptation in the humeri. To test this, Schmitt and colleagues [38] measured strain distribution on the shaft of an instrumented ‘spear’ during thrusting tasks, and demonstrated that the area of the spear nearest the trailing (dominant) hand experienced a greater portion of the force upon impact and while forcefully holding the spear against the rigid target.

Despite the evidence in support of the spear-thrusting hypothesis, other explanations for these unique humeral qualities remain viable. An unexplored suggestion made by Trinkaus and colleagues [5] is that Neandertal humeral morphology may reflect adaptation to functionally specific, and likely repetitive, tasks such as flint knapping or animal hide preparation. It is estimated that European Neandertals lived in cold conditions for many thousands of years [1], [39]. While complex physiological adaptations are probable [1], ‘clothing’ would have been necessary [39], [40]. The hides of large and small game animals would have allowed for the construction of ‘personal portable environments’ [41], providing some level of protection [42]–[44]. While the frequency of hide processing in Neandertal culture remains uncertain, one of the most ubiquitous tool types in Mousterian assemblages is the *racloir*, or side-scraper [45], [46], used to scrape sinew and hair from animal hides [47], [48]. Ethnographic evidence describes hide scraping among living groups as an arduous multi-phasic process requiring multiple hours per hide, spread over the period of a week [49]–[56]. Neandertals would have habitually knapped stone tools; nevertheless, for experienced individuals this task requires less time than hide scraping, and may impart less biomechanical strain [57], [58]. Although direct testing is required, a reasonable argument can be made that compared to hide scraping, knapping is less likely to elicit pronounced skeletal adaptations.

The spear-thrusting hypothesis is often cited as the paramount explanation for the unique humeral strength asymmetry and cross-sectional shape found in Neandertals. Arguably, further testing would help to assess whether this, or any other functional hypothesis, is the most appropriate explanation for the unique morphological characteristics of Neandertals. The forces generated through the activity of skeletal muscle (in combination with substrate reaction forces) define the functional environment believed to influence bone structure [59]. The present study takes an alternative approach to understanding how activity might have influenced bone structure by using electromyography (EMG) to quantify bi-lateral muscle activity at the chest (*pectoralis major*) and shoulder (*anterior* and *posterior deltoid*) during three distinct spear-thrusting tasks and four separate scraping tasks. This approach expands the behavioural model to include various subsistence-specific behaviours. We hypothesize that in comparison to scraping tasks, spear-thrusting tasks require greater right-side muscle activity than the left.

## Results

**Figure 1:**
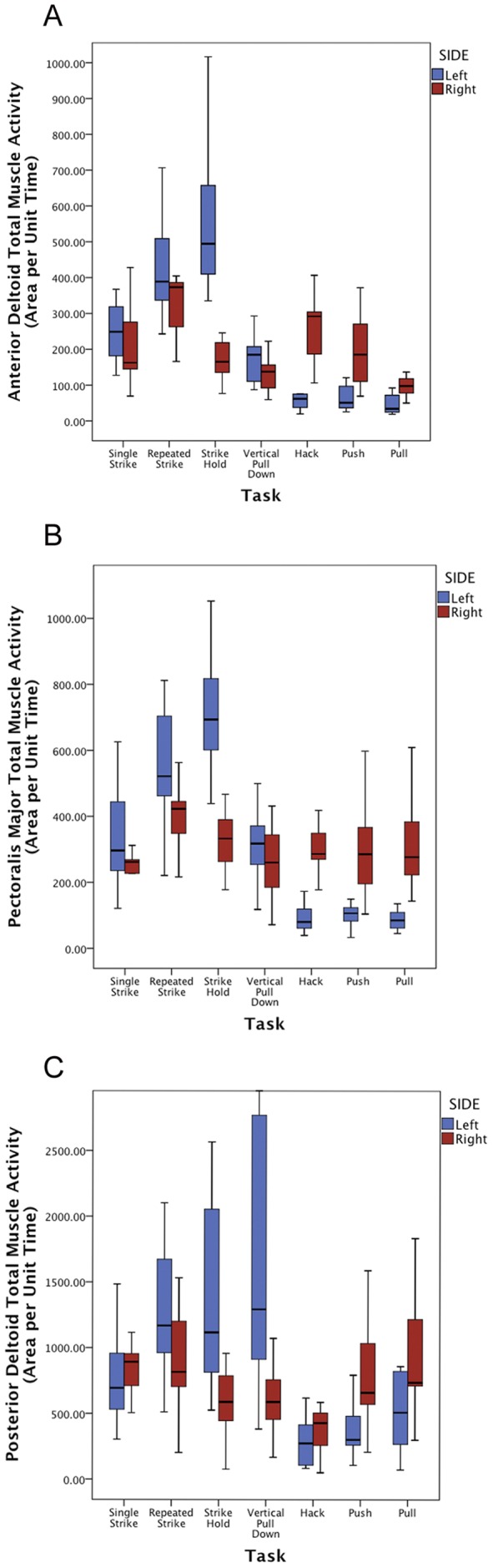
*Anterior Deltoid* (A), *Pectoralis Major* (B) and *Posterior Deltoid* (C) bilateral muscle activity for spear thrusting and scraping tasks. Total Muscle Activity (area under the curve) is presented as ‘area per unit time’, to ease interpretation.

Overall, muscle activity at the chest and shoulder reveals a dichotomous distinction between spear thrusting and single-arm scraping (Tables 1, 2, 3, Fig. 1). In contrast to predictions of bilateral muscle activity patterns required for underhand spear thrusting [17], [38], maximum muscle activity (MAX) and total muscle activity (TOT) was higher at the left (non-dominant) *anterior deltoid* (*AD*), *posterior deltoid* (*PD*) and *pectoralis major* (*PM*) compared to those same muscles on the right side of the body. Differences for MAX and TOT were significantly greater on the left side for the majority of these comparisons; this included *PM* during all three spearing activities (all values, p<.025), *AD* during strike hold (MAX p = .037 and TOT p = .0001), and *PD* during strike hold (MAX p = .049 and TOT p = .005).

**Table 1:** Bilateral *anterior deltoid* maximum (MAX) and total (TOT) muscle activity for all scraping and spearing tasks.

	MAX				TOT			
	Right	Left	p	%DA	Right	Left	p	%DA
**Spearing Tasks**								
Single Strike	678.88 (237.09)	1011.40 (563.87)	.068	−39.34	279.72 (136.60)	368.78 (171.74)	.166	−27.46
Repeated Strike	712.80 (201.01)	960.26 (472.86)	.112	−29.58	162.43 (52.74)	220.12 (98.31)	.097	−30.16
Strike Hold	586.29 (247.27)	1035.21 (580.91)	**.037**	−55.37	395.71 (167.07)	1299.97 (510.71)	**.000**	−106.65
**Scraping Tasks**								
Hack	499.77 (162.30)	107.63 (72.36)	**.000**	129.12	127.02 (43.52)	36.22 (25.85)	**.000**	111.24
Vertical Pull Down	448.05 (211.90)	448.99 (100.46)	.983	−0.21	294.65 (161.96)	377.58 (170.39)	.124	−24.67
Push	484.51 (263.72)	210.42 (153.17)	**.006**	78.88	390.96 (213.83)	170.40 (146.33)	**.009**	78.58
Pull	412.76 (126.27)	117.01 (102.98)	**.000**	111.65	201.89 (69.91)	93.37 (57.06)	**.000**	73.51

data presented as: average (standard deviation) positive values indicate higher right side muscle activity, negative values indicate higher left side activity.

**Bold**: significant difference between right and left (p ≤ 0.05), %DA: percent directional asymmetry (((right - left)/(average of left and right)) ×100); negative %DA values indicate greater left side muscle activity.

**Table 2:** Bilateral *pectoralis major* maximum (MAX) and total (TOT) muscle activity for all scraping and spearing tasks.

	MAX				TOT			
	Right	Left	p	%DA	Right	Left	p	%DA
**Spearing Tasks**								
Single Strike	808.30 (309.73)	1165.83 (592.54)	**.022**	−36.22	317.76 (87.73)	448.56 (196.78)	**.015**	−34.14
Repeated Strike	723.53 (260.77)	1138.29 (573.27)	**.009**	−44.55	181.99 (55.86)	277.94 (117.44)	**.008**	−41.72
Strike Hold	727.90 (184.44)	1216.07 (543.45)	**.008**	−50.22	701.00 (206.96)	1590.04 (407.81)	**.000**	−77.61
**Scraping Tasks**								
Hack	569.36 (147.71)	136.72 (55.80)	**.000**	122.55	148.44 (31.48)	46.64 (20.67)	**.000**	104.37
Vertical Pull Down	496.94 (165.82)	592.43 (185.21)	.088	−17.53	521.72 (223.58)	633.32 (206.10)	**.017**	−19.32
Push	621.69 (231.69)	310.01 (155.10)	**.000**	66.91	596.20 (286.53)	214.55 (97.31)	**.000**	94.14
Pull	620.48 (251.32)	179.10 (86.84)	**.000**	110.40	672.48 (334.70)	198.62 (105.05)	**.000**	108.80

data presented as: average (standard deviation) positive values indicate higher right side muscle activity, negative values indicate higher left side activity.

**Bold**: significant difference between right and left (p ≤ 0.05), %DA: percent directional asymmetry (((right - left)/(average of left and right)) ×100); negative %DA values indicate greater left side muscle activity.

**Table 3:** Bilateral *posterior deltoid* maximum (MAX) and total (TOT) muscle activity for all scraping and spearing tasks.

	MAX				TOT			
	Right	Left	p	%DA	Right	Left	p	%DA
**Spearing Tasks**								
Single Strike	1920.34 (825.33)	3488.52 (3594.85)	.111	−57.99	1094.75 (377.20)	1258.25 (196.78)	.528	−13.90
Repeated Strike	2294.96 (1110.64)	3038.32 (2742.19)	.260	−27.87	448.21 (205.79)	776.04 (686.65)	.065	−53.55
Strike Hold	1896.78 (1032.27)	3685.25 (3470.81)	**.049**	−64.08	1351.90 (798.85)	3709.22 (3022.35)	**.005**	−93.15
**Scraping Tasks**								
Hack	1056.60 (926.90)	802.41 (857.51)	.454	27.34	251.49 (227.37)	205.32 (215.08)	.597	20.21
Vertical Pull Down	1439.02 (756.86)	3475.24 (3298.61)	**.035**	−82.87	1320.18 (773.57)	3965.02 (3490.95)	**.012**	−100.08
Push	1976.00 (1177.68)	1798.15 (2236.05)	.801	9.42	1624.79 (836.60)	1294.65 (2186.43)	.602	22.62
Pull	2066.13 (1288.14)	1491.02 (1508.57)	.299	32.34	2221.55 (1504.79)	1428.44 (1424.05)	.221	43.45

data presented as: average (standard deviation) positive values indicate higher right side muscle activity, negative values indicate higher left side activity.

**Bold**: significant difference between right and left (p ≤ 0.05), %DA: percent directional asymmetry (((right - left)/(average of left and right)) ×100); negative %DA values indicate greater left side muscle activity.

Earlier research had assumed that the underhand spear thrust involved similar mechanics to the stroking of a pool cue, and thus the dominant limb would generate the majority of the force while the non-dominant limb would serve primarily as a guide [17], [38]. Higher bending forces were predicted in the trailing arm as the proximally-inserted flexors (*pectoralis major* and *anterior deltoid*) pulled the proximal aspect of the humerus forward while the resistance offered by the object being speared exerted a posteriorly directed force on the distal humerus [38]. Spear thrusting involves a tensing of the body, a transfer of weight from the back to the front foot, slight medial rotation of the hips followed by the shoulders, and a simultaneous forward thrust of the arms. It appears that because the left hand is the closest point of contact upon impact and continued pushing against the target, the majority of the substrate reaction force is translated along the spear and is countered primarily by aspects of the musculoskeletal system on the left side of the body.

During the performance of all three unimanual scraping tasks, right side MAX and TOT was significantly greater at the *AD* (all values, p<.01) and *PM* (all values, p<.02) compared to the left (Tables 1, 2, 3, Fig. 1). In keeping with this trend, muscle activity was greater at the right *PD* compared to the left, but not significantly so. In contrast, performance of the fourth scraping activity, two-handed vertical pull-down, revealed the opposite trend: TOT at *PM* (p = .017) and MAX and TOT at *PD* (p = .035 and p = .012, respectively) were significantly greater on the left side compared to the right. Although the movements involved in the push task appear biomechanically opposite to the pull and hack tasks, shoulder and chest muscle activity was similarly asymmetric and right dominant in all cases. This suggests, as one might expect, that right-handed hide preparation engenders muscular activity that loads the humeri more unilaterally towards the right side. Contrary to this, although bilateral differences were found in some cases, during two handed vertical pull-down, muscle activity on the right and left sides were more comparable than during any other scraping or spearing task.

## Discussion

The results demonstrate that during spear thrusting tasks, patterns of activity among muscles that originate at the shoulder and chest and insert onto the humerus are significantly greater on the left side of the body compared to the right. In general terms, muscular activity translates to forces imposed upon the diaphyses of long bones and can stimulate remodeling. The results suggest that if the regular performance of spear thrusting tasks caused an asymmetric increase in humeral rigidity, the left humerus would become more rigid than the right; the opposite pattern to what is found in the Neandertal fossil record. Overall, these results do not support a fundamental tenet of the spear-thrusting hypothesis; that underhand thrusting requires greater muscle activity on the right side of the body compared to the left [17], [38]. This finding contrasts with the conclusions of Schmitt et al. [38], which measured bending strain on the spear itself. It remains unclear which of these two methodological approaches (Schmitt et al. [38] vs. the present study) most accurately reflects spearing-induced osteogenesis in the arms. A study that replicates both methods is required to clarify any lack of concordance. While muscle activity *per se* is not an expression of force production, muscle activity is directly related to the production of force. Because the *deltoid* and *pectoralis* insert onto the humerus, measurements of muscle activity provide a description of the loads imposed upon the humerus. Patterns of muscle activity recorded during three separate unimanual scraping tasks revealed significantly greater right side (scraping, dominant hand) muscle activity compared to the left. This finding supports the idea that if scraping tasks were a regular construct of Neandertal life, as is the case in modern human groups that process hides [49], [52], these activities may be a viable alternative for explaining the pronounced bilateral humeral diaphysis strength asymmetry found in Neandertals.

These analyses raise a number of important questions that require further testing. First, do the patterns of asymmetric muscle activity measured during spearing and scraping tasks accurately reflect patterns of bone strain, and ultimately bone adaptation? Second, while it is plausible that the magnitude of bone strain resulting from intense spear thrusting could cause adaptation in humeral structure, is the same true for a less-intense, yet highly repetitive, activity such as scraping? For practical and ethical reasons, the first of these questions is difficult to directly test in humans (for a rare example see [60]). Nevertheless, while strain magnitude may be a primary influence [61] experimental work involving animal models suggests that the number of strain cycles (repetitions) also plays a critical role in the mechanism by which bone responds to mechanical strain [62]–[64]. Thus, if scraping tasks were adequately strenuous and performed regularly this activity may stimulate adaptive bone remodeling. Although we cannot be sure of the precise hide preparation techniques used by Neandertals, ethnographic accounts can provide useful models. Hide-processing tasks performed by the Inuit are described as arduous, highly technical, and time-consuming [49], with experienced individuals needing more than 8 hours spread over several days to make a dried skin suitable for clothing. Ethnographic reports of the Wolayta and Oromo of Ethiopia agree with this timeline and estimate that six and ten hours, respectively, are required to process a single cattle hide [55], [56]. Among the Tahltan of Northern British Columbia each family processes ∼30 new hides every 12 months, effectively engaging a single individual for over half the year [52]. Although hide processing methods vary globally and throughout time, wet-hide defleshing and multiple bouts of dry-hide scraping are always involved [49]–[54]. For example, for each hide, the Inuit perform three separate scraping tasks totaling more than six hours of strenuous labour [49]. Among the Aleut of Alaska and the Tchotchke of Siberia, most hide-processing tasks are performed by females [65], [66]. Humeral bilateral strength asymmetry appears much lower in Neandertal females compared to males [17], [67], and may reflect a sexual division of labour. However, due to the dearth of paired humeri attributable to female Neandertals, the attribution of differences in sex-specific bi-manual task specialization between Neandertals and *H. sapiens* remains speculative. Additionally, it has been suggested that anterior tooth wear in Neandertals may reflect the use of the mouth as a ‘third hand’ during hide scraping and food processing [68], [69], nevertheless, there is variable evidence as to how important this action may have been [70].

Stone tool types coupled with analyses of use-wear patterns can provide insight into the subsistence activities of prehistoric groups [71]–[73]. Scrapers are the most common artefact type found in Neandertal tool assemblages of Western Europe and the Near East. The most common scraper types within Mousterian toolkits are the simple, but distinctive, retouched tool forms called *racloirs*, or side-scrapers [45], [74]. Microscopic analysis of Mousterian scrapers support the view that use is mainly confined to retouched edges, although there is little support for an association between scraper types and particular functions [48], [75]. The function of scrapers has, in fact, been shown to be variable, and includes the scraping of plants, hide, antler or bone to scraping, cutting and slicing of wood and meat or skin [48], [76]. Hide-working is typically identified as a significant scraper function, recently exemplified by the analysis of scrapers from the Mousterian site of Payre (Ardeche, France) which show polish and striations from scraping, as well as skin and hair fragments [47].

Alternatively, one might hypothesize that Neandertals spear thrusted using a left-handed grip (right hand forward), which would generate greater right side muscle activity and therefore a greater load on the right humerus. All participants in the present study were right handed, were given no instructions regarding hand placement on the spear, and all performed the tasks using the same grip: left hand towards the front of the spear with the thumb facing forward, right hand positioned at the back (for corroboration see [38]). Thus, for Neandertals to employ a ‘left-handed’ grip on the spear they would almost certainly have been left-handed. Globally, ∼90% of all humans can be classified as right-handed [77], [78], a trend that appears consistent for at least ten thousand years [79], and perhaps as far back as 500K [68], [80] or even 2 million years [81], [82]. If the rate and distribution of handedness in *Homo sapiens* is indicative of handedness distribution in Neandertals it is quite unlikely that all Neandertals in the fossil record (where matching right and left humeri are available) were left-handed and employed a left-handed spearing grip.

Finally, the results from the present study do not directly inform the hypothesis that spear thrusting is responsible for the pronounced diaphyseal shape (anteroposterior strengthening) in both the right and left humeri of Neandertals [38]. Alternative hypotheses, however, do stem from the finding of somewhat comparable muscle activity in the right and left shoulder and chest during two-handed ‘pull down’ scraping tasks. Previous research has argued that the habitual performance of repetitive bimanual tasks, such as the use of digging sticks by Later Stone Age South African women, can stimulate pronounced bilateral A-P strengthening at the humeral diaphysis [83]. Overall, it is not inconceivable that 1) the pronounced humeral strength asymmetry characteristic of Neandertals resulted from repetitive unimanual scraping tasks, and 2) that the corresponding A-P strengthened shape of the Neandertal humeral diaphysis resulted from the habitual performance of a separate scraping task(s) that was symmetric, bimanual, and loaded the humerus in the A-P plane.

In conclusion, the consistency of the results provides evidence that scraping activities, such as hide preparation, may be a key behaviour in determining the unusual pattern of Neandertal upper limb morphology. The muscle activity required to perform various underhanded spearing tasks does not lend itself to explaining the pronounced right dominant strength asymmetry found in Neandertal humeri. Future studies of spear thrusting mechanics might evaluate the action at specific joints by comparing the timing of muscle activation at the shoulder flexors, elbow flexors, and rotator cuff using EMG and motion analysis. Although it still remains to be demonstrated conclusively, if both unimanual and bimanual hide scraping tasks were performed habitually, the muscle activity associated with these tasks may be a parsimonious explanation for the Neandertal’s unique humeral morphology. Overall, these results yield important insight into the Neandertal behavioural repertoire that aided survival throughout Pleistocene Eurasia.

## Materials and Methods

### Participants

Thirteen right-handed men (age: 28 ± 6.4 years, mass: 78.0 ± 10.4 kg, height: 180.8 ± 8.9 cm) with no known musculoskeletal disorders volunteered in this experiment. Participants were prescreened to exclude any predominantly left handed individuals; handedness was determined by a brief survey adapted from Oldfield [84]. The Institutional Review Board of The Pennsylvania State University approved this study’s protocol, and all participants provided informed written consent prior to participation. The participant pictured in Figures S1, S2, S4, S5 and S7 has given written informed consent, as outlined in the PLoS consent form, to publication of their photograph.

### Electromyography

Two 2.22 cm square silver-silver chloride electrodes (Vermed Inc. Bellows Falls, VT) were placed on the right and left *anterior deltoid* (AD), *posterior deltoid* (PD), *infraspinatus* (IS), and *pectoralis major* (PM) muscles. A ground electrode was placed on the left acromion process. All sites were abraded and wiped with alcohol prior to electrode placement. Electrode placement (Fig. S1) was determined based on anatomical landmarks and verified through a series of functional tests [85]. All data were collected at 1000 Hz with an amplifier hardware system (Bortec Octopus, Calgary, Alberta, Canada) using Evart software (Version 3.21, Motion Analysis Corp., Santa Rosa, CA). EMG provides a measure of the neural excitation of a muscle, which is correlated with the production of muscle force. This study reports measures of both maximum muscle activity (MAX), a measure of the maximum integrated muscle activity (where activity is the electrical potential across the region of the muscle), and total muscle activity (TOT), a measurement of the area under the curve of the linear envelope.

### Experimental Protocol

Participants performed three different spear thrusting tasks and four different scraping tasks. The order of all tasks was randomized, and each activity was performed to the beat of a 120 Hz metronome to standardize timing across participants. Every fourth beat (i.e., every two seconds) produced a unique tone allowing participants to synchronize their movements in multiples of 0.5 seconds or 2.0 seconds, as per the desired protocol. Participants performed several practice trials of each task to ensure their comfort with the frequency and effort necessary for each task (further detail below). Three 12-second trials of each task were recorded. All participants first performed a baseline task for the purpose of normalizing the EMG data. This baseline task required the participants to stand in an anatomically neutral position holding a 2.25 kg plate in each hand. Participants were instructed to then flex both the shoulder and elbow in the sagittal plane to 90°. The participants then began a sequence of externally and internally rotating the shoulder 90° in 2 second intervals, for a total of 18-seconds. Elbow flexion was maintained at 90 degrees of flexion throughout.

### Spearing Tasks

Spearing protocols were adapted from Schmitt and colleagues [38]. Each spearing task was performed with a wooden dowel (length  = 214 cm, diameter  = 3.5 cm) with a rounded end. Participants were instructed to perform each spearing tasks using an underhanded grip, at a self-selected distance from the target. The ‘target’ was composed of five 61×61 cm pieces of carpet (thickness  = 3.5 cm), placed in front of a fitness mat (thickness  = 60 cm). The target was located approximately 100 cm above the ground (Fig. S2). Although not explicitly instructed, all participants chose to operate the spear leading with the non-dominant limb (see Discussion). Additionally, while thrusting the spear, participants were instructed to exert maximal effort, striking the mat with as much force as possible. Participants were required to keep their feet stationary on the ground, but no further limitations were imposed upon the participants body position.

Three different spearing tasks were performed:

#### Single strike

A single thrust, followed by a rapid withdrawal, contacting the target in 2-second intervals.

#### Repeated strike

Three consecutive rapid strikes performed at an interval of 0.5 seconds, followed by a 2.5 second rest period.

#### Strike hold

Single strike with continued isometric drive of spear into the target for 2 seconds, followed by a 2 second withdrawal resting period.

### Scraping Tasks

All scraping tasks were performed on a 61 cm×61 cm piece of carpet, with the fibers facing out. Carpet was used as a substitute for large ungulate hide; the regularity of the carpet’s material properties ensured that the scraping surface was consistent for each participant. It was explained to the participants that the softer carpet fibers represented material that needed to be removed (e.g., sinew, adipose tissue) during the scraping process. Participants were instructed to remove carpet fibers within a relatively narrow strip (5 cm×30 cm) during each 12-second trial, and to select an angle and amount pressure so that the under layer of the carpet was not damaged. Thus, the intensity (downward pressure applied) was relatively consistent amongst the participants. The four scraping tasks performed were chosen to simulate specific movements used by modern hide processing groups using non-mechanised tools. Arguably, these tasks are variations of tasks also performed in prehistory. These include:

#### Hack

The *hack* was performed with the dominant hand while grasping a chisel (FatMax 1” width, Stanley Black & Decker Inc., New Britain, CT) wrapped in athletic tape (Fig. S3). Participants stood in an athletic stance facing the target, and maintained their balance with the non-dominant arm. The target carpet was positioned near shoulder height, and was fixed to a wooden ramp, which was angled away from the participant at 15°. The participant then performed a repeated downward striking motion in the sagittal plane contacting the target every 0.5 seconds (Fig. S4).

#### Vertical pull down

This task was performed with both hands, while standing. A mini-crowbar (Wonder Bar, Stanley Black & Decker Inc., New Britain, CT) was used to simulate an end-scraper (Fig. S3). Participants were instructed to hold the bottom of the simulated scraper with the dominant hand and to apply additional pressure as needed to remove the carpet fibers with the non-dominant hand placed on top of the tool (Fig. S5). The range of motion began at the participants shoulder and ended at the mid-abdomen. The start of the task began with the tool placed on the carpeted target near the level of the participants shoulder; this was followed by a downward scrape in 2-second intervals.

Push and Pull: These tasks were performed with the participant in a kneeling bent-over position, using a replica Mousterian side-scraper (Fig. S6). Depending on which task was being performed, the participant began by either scraping away from the body (*push*) or scraping toward the body (*pull*). Each scraping action involved moving the tool in the sagittal plane, and was performed throughout a 2 second interval. For these tasks, the participant was instructed to support their body with the non-dominant limb by placing it on the scraping surface (Fig. S7).

### Data Analysis

All EMG data were first normalized to the mean of the baseline task. The normalized data were high-pass filtered (20 Hz), full-wave rectified, and the resulting linear envelope was smoothed with a low pass filter (2 Hz) using MATLAB (Version R2009a, The Mathworks Inc, Natick, MA).

### EMG Signal Identification and Isolation

In order to isolate the EMG data during the period of action for each repetition of each task, *activity burst time windows* were defined in a unique manner for each task. For all spearing trials, the processed EMG data for the right *infraspinatus* was utilized for defining *activity burst time windows* in the following ways:

#### Single strike

The peak value for each local maximum was determined. Following this, the times at which 20% of this maximum occurred (one before and one after the peak) were found. A range of 0.5 seconds before and after each of these values was considered the outer limit of the activity burst window (Fig. S8, top).

### Repeated Strike

A primary window surrounding the three characteristic muscle activity peaks was considered to start and end 0.1 seconds before and after the first and third peaks, respectively. This primary window was divided by three, and these were considered the time window for each of the three individual bouts (Fig. S8, middle).

#### Strike hold

The peak value for the period of activity was determined. Following this, the times at which 40% of the maximum (before and after the peak) were found. The window of activity was considered to start 0.225 seconds before the first instance and end 0.225 seconds after the second instance of 40% of the maximum. These values were all self-selected in an attempt to capture all muscle activity, and minimize the presence of periods of rest (*i.e.*, little to no muscle activity), within the *activity burst time window*. Once these time windows were determined for the right *infraspinatus*, they were applied across all eight muscles and were visually inspected to ensure that all relevant data had been isolated (Fig. S8, bottom).

Scraping tasks were processed in a different fashion, as generally there were no significant periods of rest between bouts of activity. A peak detection algorithm was used to find all local maxima and minima for each trial of activity (Fig. S9). For each scraping task, an appropriate muscle was selected, based on visual inspection, for peak detection (*hack* – Right *anterior deltoid*; *vertical pull down* – Left *posterior deltoid; push* & *pull* – Right *posterior deltoid*). Activity burst time windows were defined as the time from one local minimum to the adjacent minimum. Again, these windows were applied to all eight muscles and visually inspected for accuracy. If a time window did not fit a pre-determined range, as decided based on metronome timing, these data were removed from future analyses; *e.g.*, if a *hack* activity burst time window was found to be 1.1 seconds, this was removed, as it is likely that a minimum was missed by the peak detector (as per the *hack* protocol, activity time bursts should be close to 0.5 seconds). This type of erroneous data could also potentially be due to incorrect participant timing during trials. This exclusion ensured that homologous data windows were compared.

Once the *activity bursts* were defined for each muscle and condition, the peak (maximum muscle activity; MAX) and area under the curve (total muscle activity; TOT) was calculated. MAX and TOT were used for intra-participant comparisons of muscle activity (i.e., left versus right). Since all EMG traces were normalized to the baseline task, values of MAX are unitless (reported as mV/mV). As a result, units for TOT are reported in seconds (mV*s/mV). Since EMG was normalized to a standard baseline task, opposed to maximum voluntary contraction, the magnitudes of MAX and TOT represent the ratio of the muscles activity of the task to the baseline. MAX and TOT are simply used for comparison between right and left values of the same muscle during the same activity. Averages for both MAX and TOT variables were computed from all activity bursts over the three total trials for each task.

### Statistical Analyses

Student’s paired t-tests were performed to compare right vs. left maximum muscle area (MAX) and total muscle area under the curve (TOT). All statistical comparisons were performed using PASW (Version 18.0.0, IBM Corp., Armonk, NY). Tests were considered statistically significant at p ≤ 0.05.

## Supporting Information

Figure S1 **Electrode placement.** Left to right: left *posterior deltoid*, left *infraspinatus*, right *infraspinatus*, right *posterior deltoid*, right *anterior deltoid*, right *pectoralis major*, left *pectoralis major*, left *anterior deltoid*.
(TIF)

Figure S2 **Experimental set up for spearing tasks.**
(TIF)

Figure S3 **Chisel (top) and mini-crowbar utilized in the** ***hack*** **and** ***vertical pull down*** **scraping tasks, respectively.**
(TIF)

Figure S4 **Experimental setup for the** ***hack*** **task**.
(TIF)

Figure S5 **Experimental setup for the** ***vertical pull down*** **scraping task.**
(TIF)

Figure S6 **Replica side scrapers used for the** ***push*** **and** ***pull*** **scraping tasks.**
(TIF)

Figure S7 **Experimental setup for the** ***push*** **and** ***pull*** **scraping tasks.**
(TIF)

Figure S8 **Activity window definition; spearing tasks.** Processed EMG patterns shown in black, with the final activity time windows shown in green.
(TIF)

Figure S9 **Activity window definition; scraping tasks.** Processed EMG patterns in black. Blue lines indicate local minima, determined from peak detection code, whereas green lines illustrate the activity bursts.
(TIF)
