# Supplementary figures and images for: Neandertal Humeri May Reflect Adaptation to Scraping Tasks, but Not Spear Thrusting
Source: PLoS One. 2012 Jul 18;7(7):e40349. doi: 10.1371/journal.pone.0040349 (PMC3399840; doi:10.1371/journal.pone.0040349)

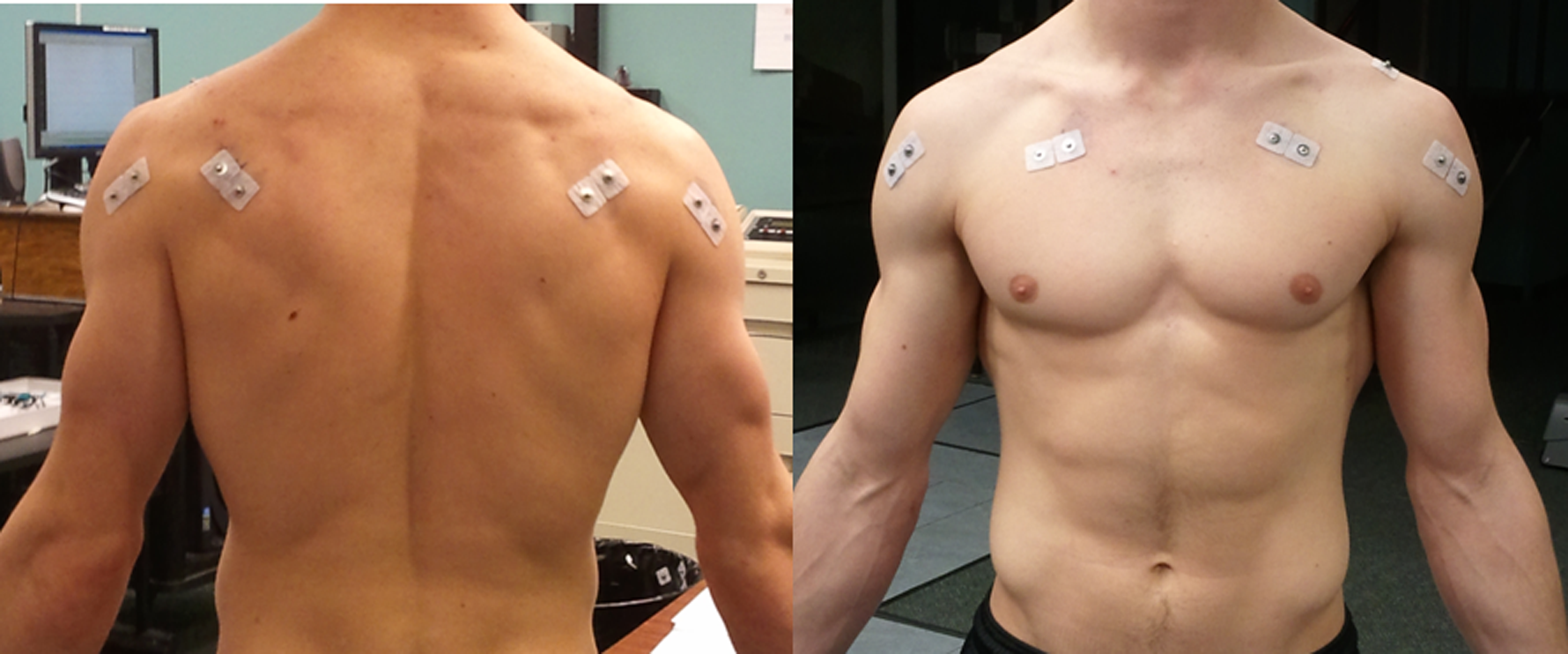

Supplement: Figure S1 — Electrode placement. Left to right: left posterior deltoid, left infraspinatus, right infraspinatus, right posterior deltoid, right anterior deltoid, right pectoralis major, left pectoralis major, left anterior deltoid. (TIF) [file pone.0040349.s001.tif]

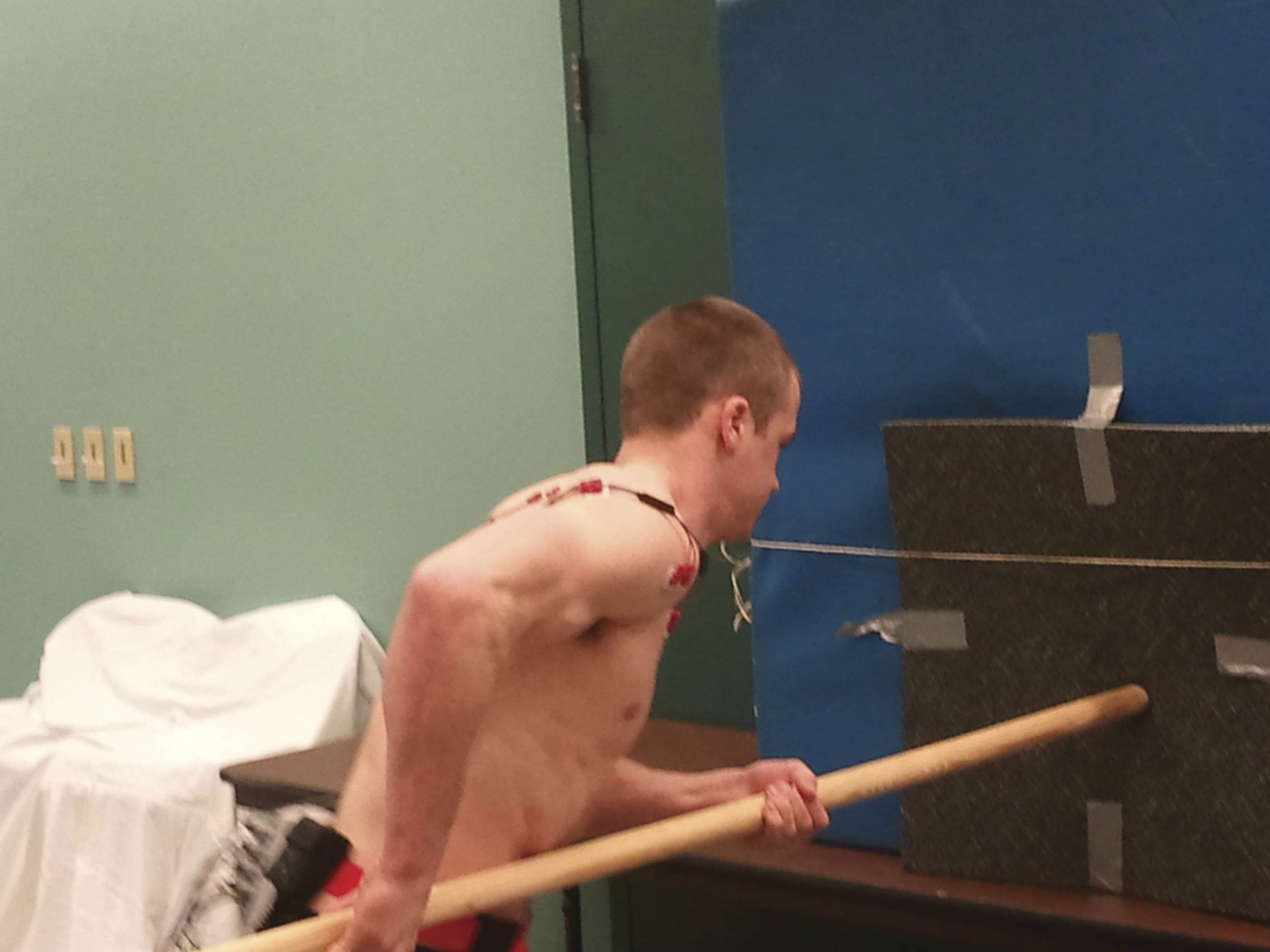

Supplement: Figure S2 — Experimental set up for spearing tasks. (TIF) [file pone.0040349.s002.tif]

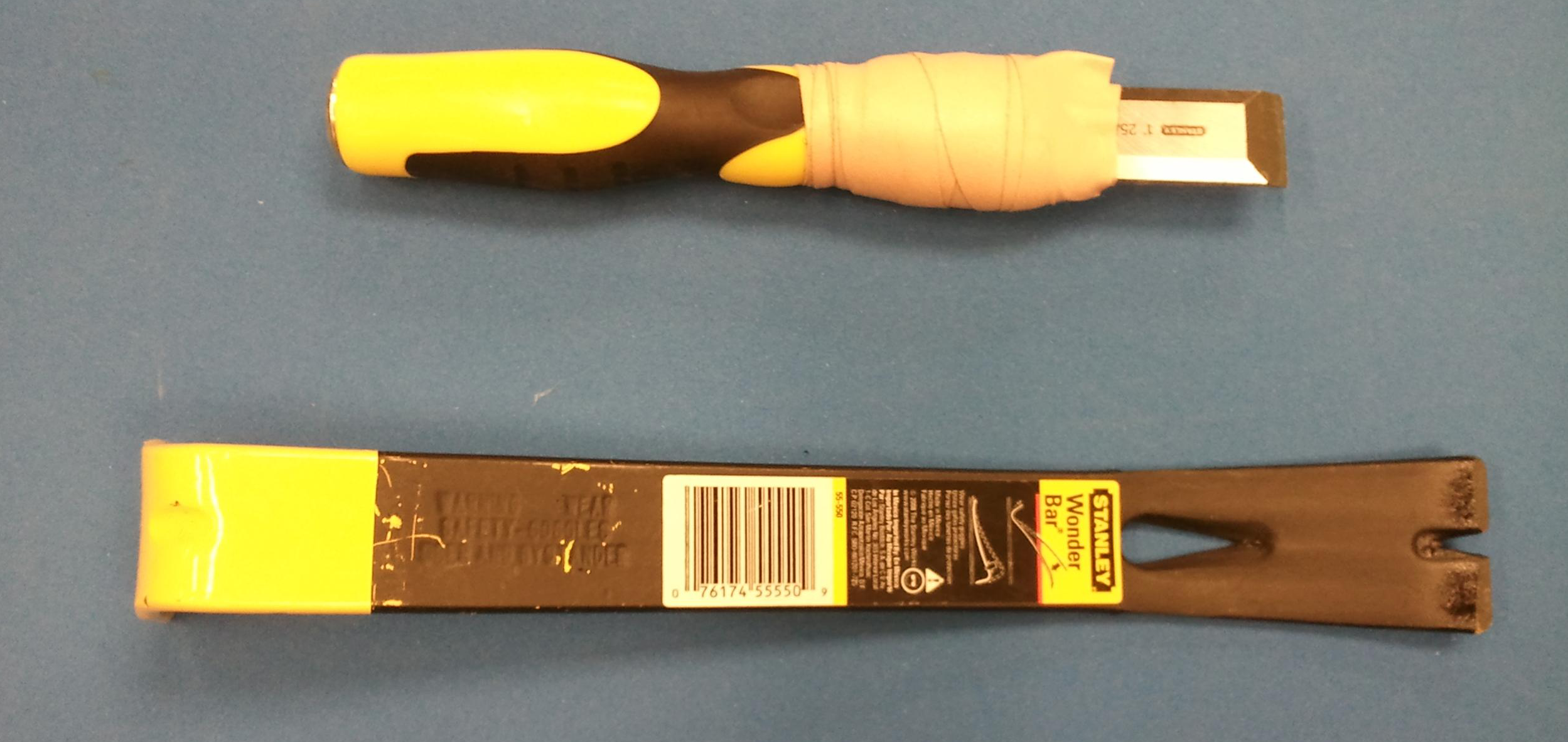

Supplement: Figure S3 — Chisel (top) and mini-crowbar utilized in the hack and vertical pull down scraping tasks, respectively. (TIF) [file pone.0040349.s003.tif]

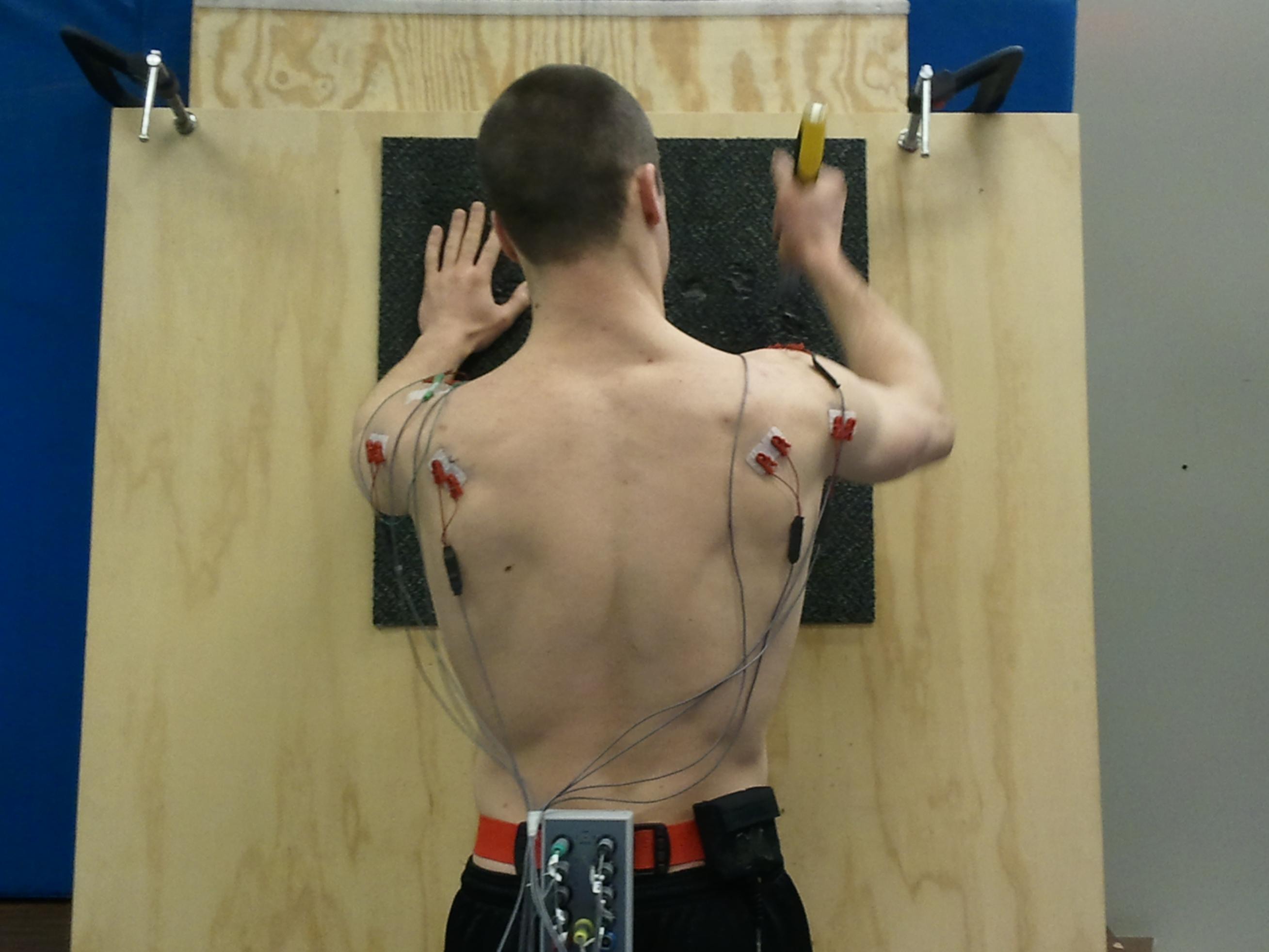

Supplement: Figure S4 — Experimental setup for the hack task. (TIF) [file pone.0040349.s004.tif]

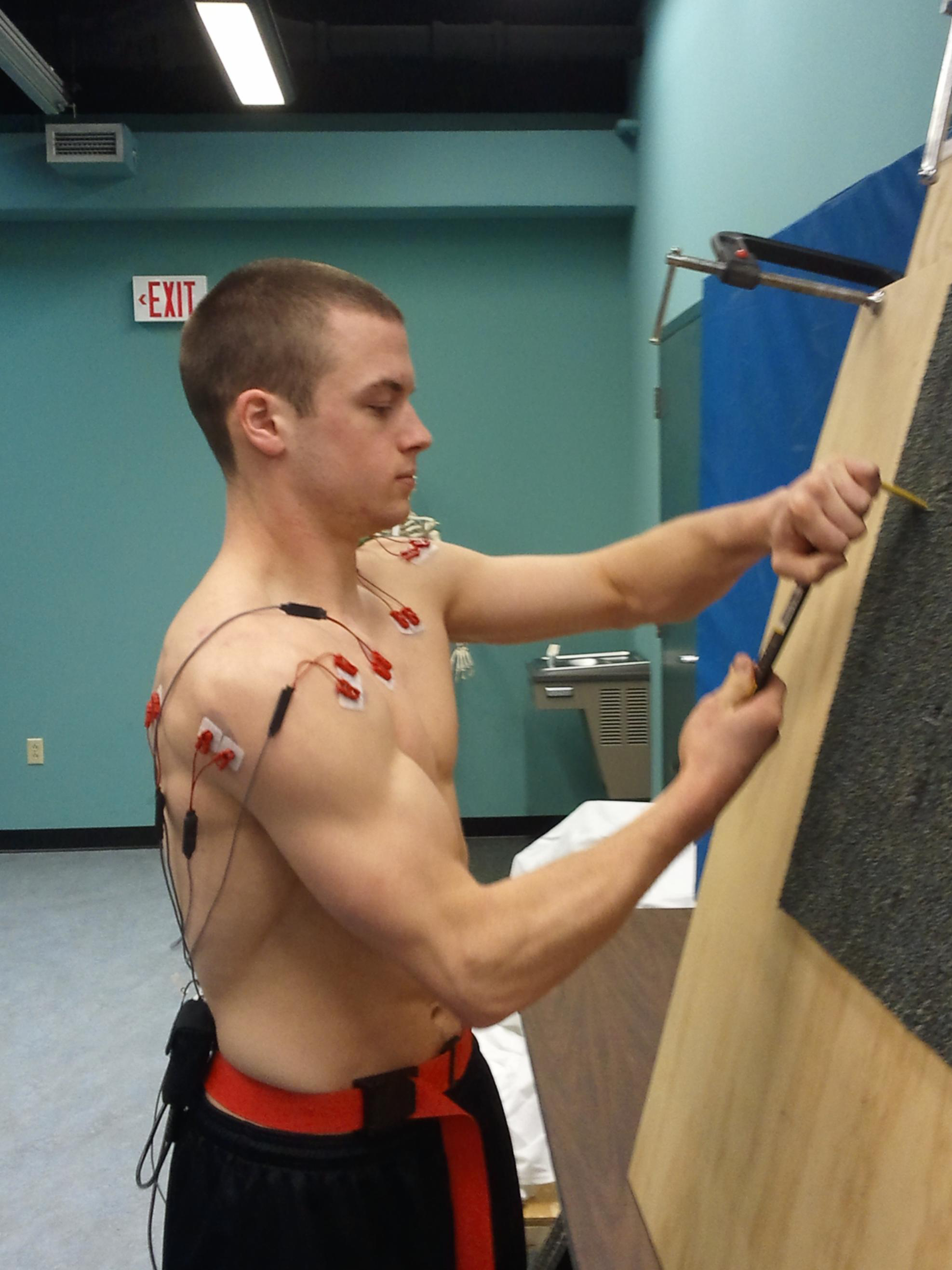

Supplement: Figure S5 — Experimental setup for the vertical pull down scraping task. (TIF) [file pone.0040349.s005.tif]

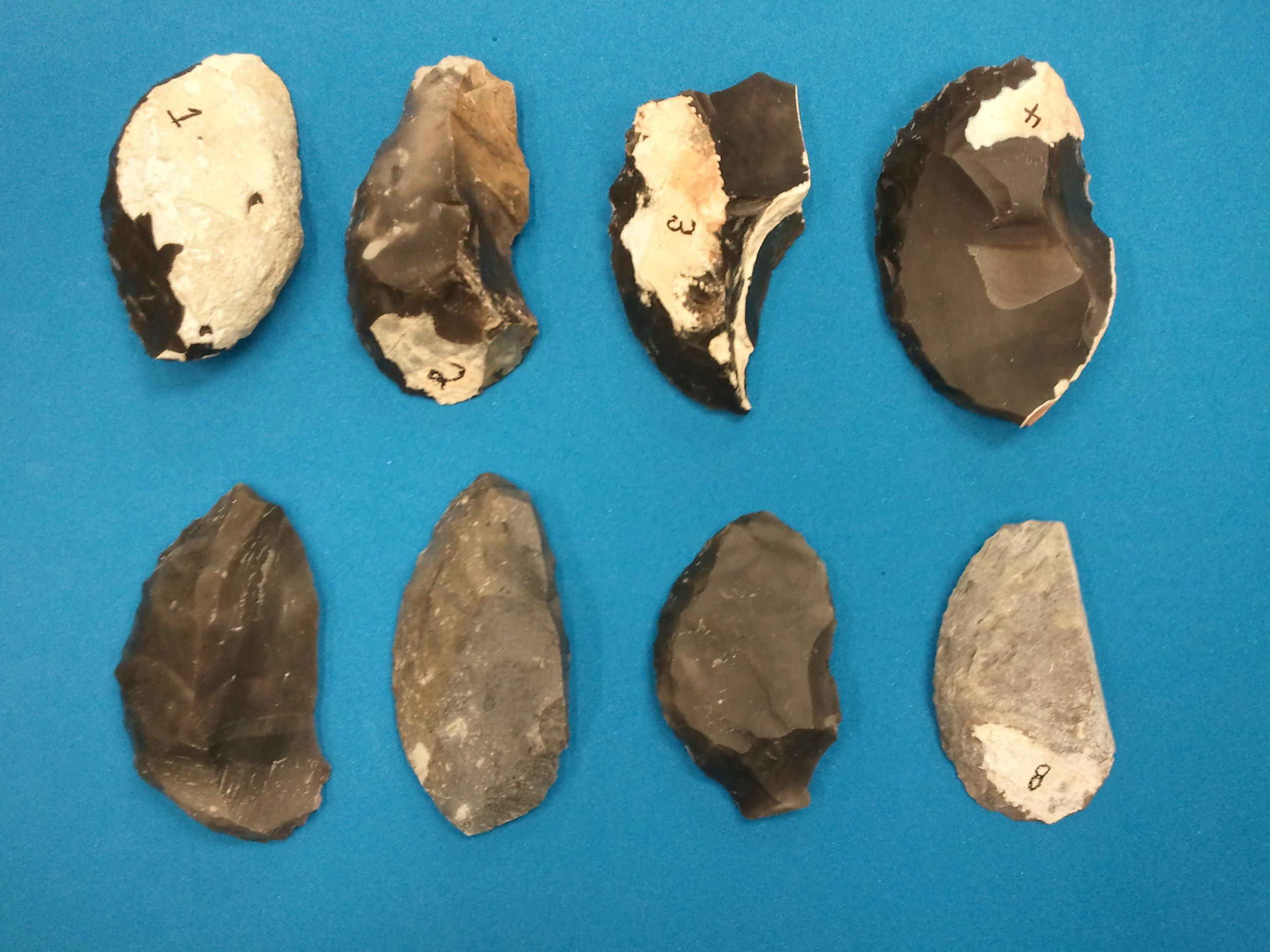

Supplement: Figure S6 — Replica side scrapers used for the push and pull scraping tasks. (TIF) [file pone.0040349.s006.tif]

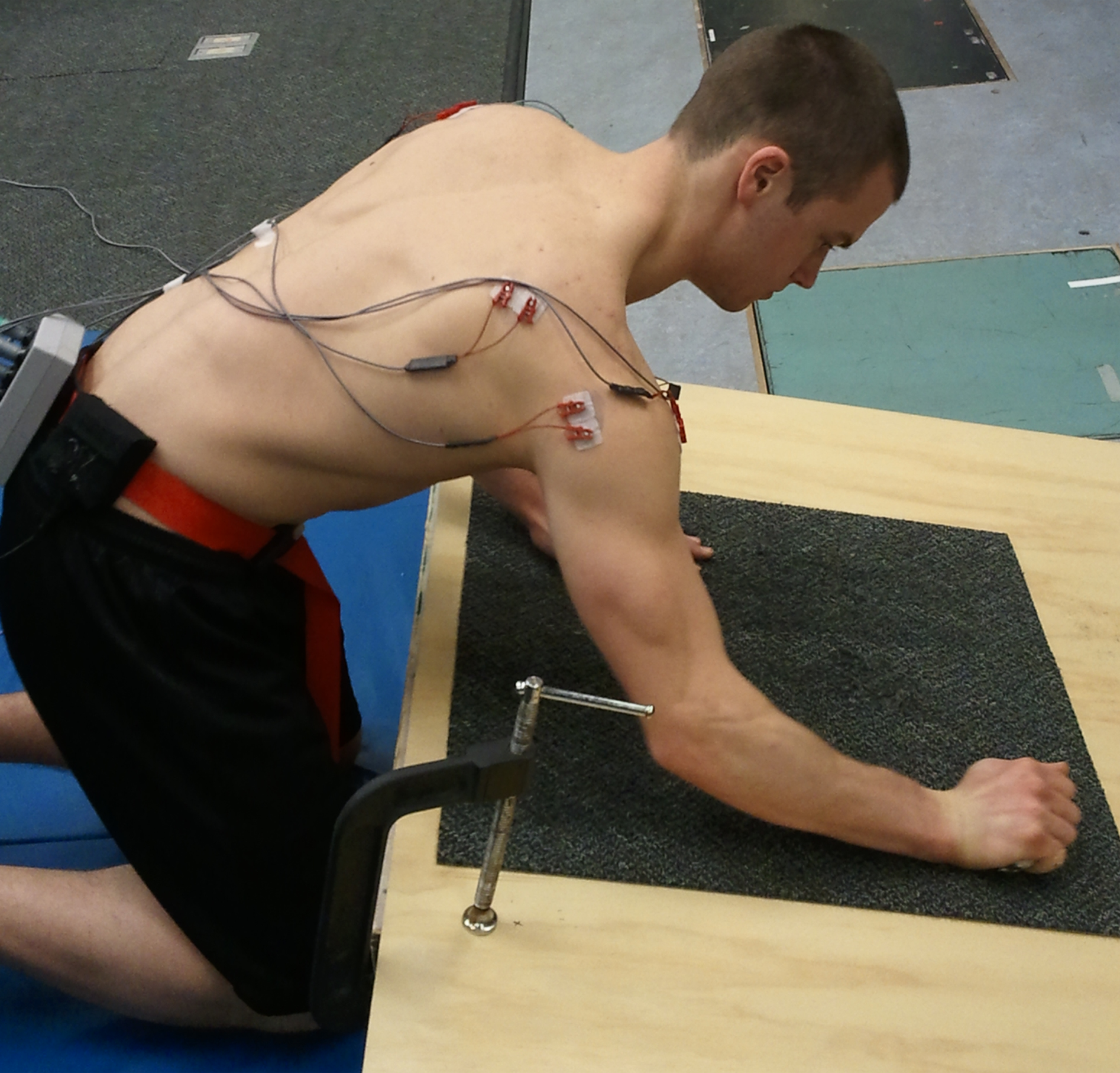

Supplement: Figure S7 — Experimental setup for the push and pull scraping tasks. (TIF) [file pone.0040349.s007.tif]

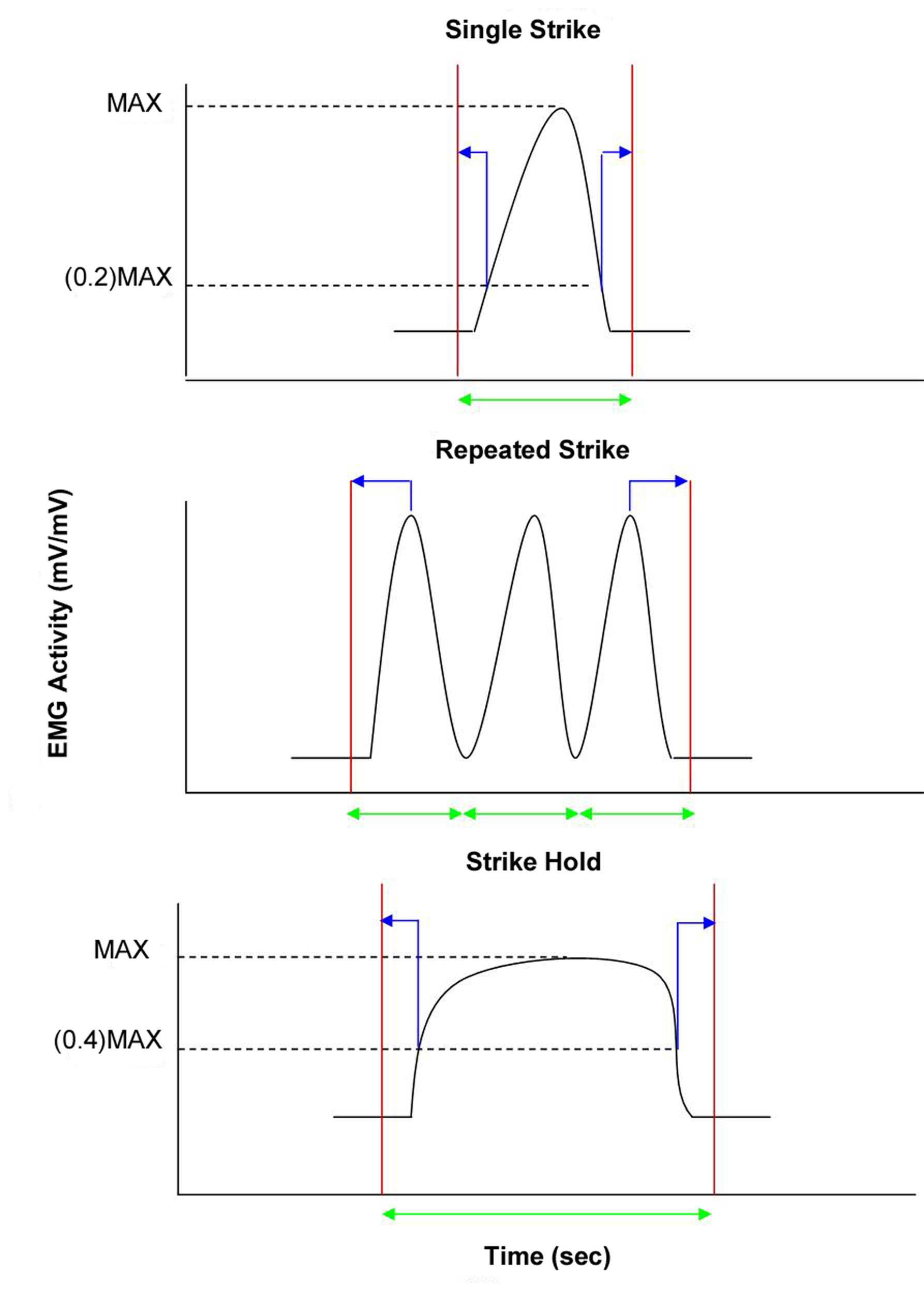

Supplement: Figure S8 — Activity window definition; spearing tasks. Processed EMG patterns shown in black, with the final activity time windows shown in green. (TIF) [file pone.0040349.s008.tif]

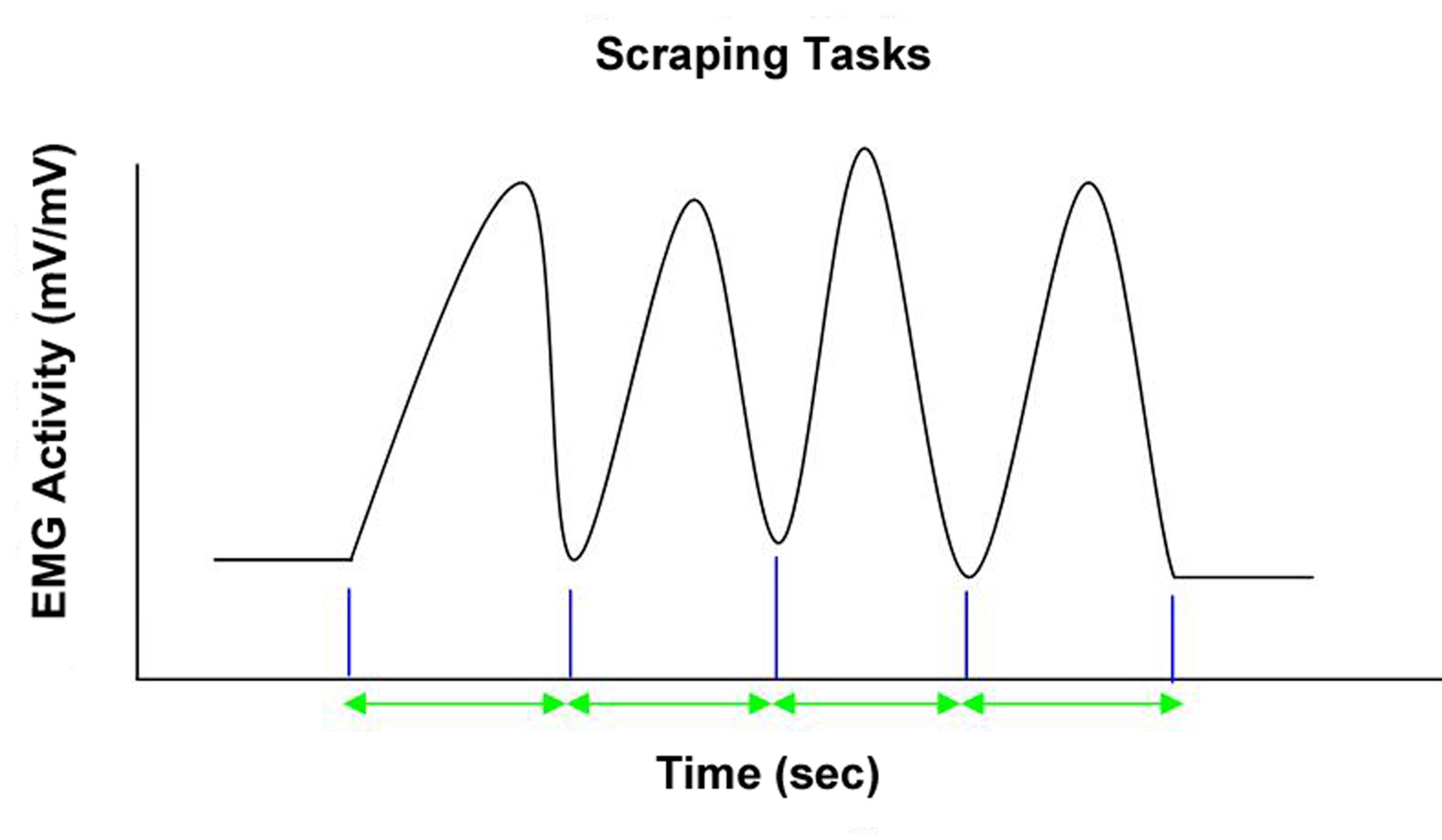

Supplement: Figure S9 — Activity window definition; scraping tasks. Processed EMG patterns in black. Blue lines indicate local minima, determined from peak detection code, whereas green lines illustrate the activity bursts. (TIF) [file pone.0040349.s009.tif]
